# The effectiveness of a school-based, universal mental health programme in six European countries

**DOI:** 10.3389/fpsyg.2022.925614

**Published:** 2022-08-08

**Authors:** Carmel Cefai, Liberato Camilleri, Paul Bartolo, Ilaria Grazzani, Valeria Cavioni, Elisabetta Conte, Veronica Ornaghi, Alessia Agliati, Sabina Gandellini, Sanja Tatalovic Vorkapic, Maria Poulou, Baiba Martinsone, Ieva Stokenberga, Celeste Simões, Margarida Santos, Aurora Adina Colomeischi

**Affiliations:** ^1^Department of Psychology, Centre for Resilience and Socio-Emotional Health, University of Malta, Msida, Malta; ^2^Department of Statistics and Operations Research, University of Malta, Msida, Malta; ^3^Department of Psychology, University of Malta, Msida, Malta; ^4^“R. Massa” Department of Human Sciences for Education, University of Milano-Bicocca, Milan, Italy; ^5^Faculty of Teacher Education, University of Rijeka, Rijeka, Croatia; ^6^Department of Educational Sciences and Early Childhood Education, University of Patras, Patras, Greece; ^7^Department of Psychology, University of Latvia, Riga, Latvia; ^8^Department of Education, Social Sciences and Humanities, University of Lisbon, Lisbon, Portugal; ^9^Faculty of Sciences in Education, Stefan cel Mare University, Suceava, Romania

**Keywords:** mental health, social and emotional learning, universal intervention, school-based programme, effectiveness, *PROMESH*

## Abstract

As children and young people today face ever increasing social, emotional and mental health challenges, schools, as one of the primary systems in children’s lives, are called to broaden their agenda and help to address these challenges. This paper discusses the evaluation of a school-based, universal mental health promotion programme developed recently for the European context. The programme provides a universal curriculum from early years to high school, aiming to promote social and emotional learning and resilience and prevent social, emotional, and behavioural problems in children and adolescents. A total of 7,789 students (and their teachers and parents) from kindergarten to high school across 6 countries in Europe were recruited from 434 classrooms in 124 schools, making use of cluster sampling. A quasi-experimental longitudinal design was used to evaluate the effectiveness of the programme on students’ outcomes by comparing the groups’ outcomes within times (pre-test vs. post-test) and between groups (experimental vs. control group). A total of 779 classroom teachers completed pre-and-post scales measuring students’ social and emotional learning, mental health and academic achievement. Results indicate that the experimental group had significantly larger increase in social and emotional competence and prosocial behaviour, and a decrease in mental health issues (externalising and internalising problems). No significant impact was found for academic outcomes. The findings are discussed in view of the limitations of the study and areas for further research.

## Introduction

The current challenges taking place in various parts of the globe, such as the COVID-19 pandemic, displacement and forced migration due to war and conflict, and climate change, have exacerbated the mental health issues faced by children and young people. The pandemic has led to an increase in mental health difficulties, particularly amongst those already at risk (OECD, 2020a; United Nations Educational, Scientific and Cultural Organisation [UNESCO], 2020) and underlined the opportunities for schools to address these issues (Norwich et al., 2022). Between ten to twenty percent of school children experience mental health issues during their school years, with half of the mental health problems developing before the age of fourteen (WHO Regional Office for Europe, 2018). In a recent meta-analysis of 192 epidemiological studies, Solmi et al. (2022) reported that the proportion of young people who experienced the onset of mental health issues before the ages of 14, 18, and 25 years, was 35, 48, and 63%, respectively, with the peak age being 14.5 years. Depression and anxiety are among the top five causes of illness, with 14% of adolescents manifesting diagnosable depressive symptoms before the age of 18, whilst suicide is the leading cause of death among adolescents in low- and middle-income countries and the second leading cause of death in high-income countries (WHO Regional Office for Europe, 2018; WHO, 2021). Mental health issues amongst children account for 13% of the global burden of disease in children (WHO, 2021), which is set to increase in the coming decades (Baranne and Falissard, 2018). The latest Health Behaviour in School-aged Children Survey by WHO (Inchley et al., 2020) reports that the positive gains in the various aspects of adolescent health since the previous study 4 years ago, were accompanied by an overall decline in mental and social wellbeing. Students’ responses indicated a decrease in liking for school, an increase in academic pressure, and lack of supportive environments at school. Students from low socio-economic status had lower levels of support from school peers and friends. Another recent study reported that one in five of school children in Europe are unhappy and anxious about the future as a result of bullying, academic pressure and loneliness (UNICEF and the European Union, 2021).

As children and young people face these ever increasing social and emotional challenges, there is a more urgent call for schools, as one of the primary systems in children’s lives, to go beyond narrow sectoral goals and help to address these challenges (WHO, 2020). Schools have access to most children and young people for a considerable time during a critical period in their cognitive and social and emotional development. WHO (2017) recommends that schools function as one of the primary mental health support systems for students, providing a broad spectrum of mental health actions that encompass promotion, prevention, intervention, and rehabilitation. The WHO’s Global School Health Initiative (WHO, 2020) emphasises the importance of focusing on and enhancing the psychosocial environment in schools with a view to fostering emotional and social wellbeing.

The promotion of mental health and wellbeing in school, however, is still not widely recognised as one of the main objectives of education. Academic achievement is still seen as the primary measure of success in many educational systems across the world as well as in international assessments of cognitive abilities (OECD, 2020b). In a survey with 1,500 schools from 10 European countries, Patalay et al. (2016, 2017) found that only 47% reported that mental health provision was a high priority, whilst more than half did not implement a mental health school policy. Most support was dedicated to students with learning disabilities (78%) and students with mental health problems (66%) rather than to the prevention of problems (55%) or promotion of student wellbeing (50%).

There is increasing evidence that universal interventions to promote mental health and wellbeing in school through curricular and systemic interventions, are effective in supporting the mental health and wellbeing of children and young people (Durlak et al., 2011; Weare and Nind, 2011; Goldberg et al., 2019; OECD, 2021). In a seminal review of studies on social and emotional learning in schools with over 200 studies, Durlak et al. (2011) reported that universal SEL interventions led to an increase in social and emotional competence, positive attitudes, prosocial behaviour and academic performance, and to a decrease in mental health issues such as anti-social behaviour, substance use, anxiety and depression. Research also indicates the importance of school-based mental health interventions as an effective strategy to prevent the onset of mental health issues during adolescence (Stockings et al., 2016; Woods and Pooley, 2016). A recent OECD report on the implementation of school based SEL interventions in ten major cities across the world, found that interventions were strongly related to psychological wellbeing as well as academic achievement amongst 11 and 15 year-old children. The strength of the interventions was particularly related to their capacity to shape behaviour and lifestyles and to better leverage cognitive capabilities (OECD, 2021).

This paper focuses on mental health in schools in terms of the promotion of mental health as defined by WHO (2018), namely a state of wellbeing where one realises his or her own abilities and can cope with the normal stresses of life, with a positive sense of identity, an ability to manage thoughts and emotions, to build social relationships, and to acquire an education that allows active citizenship as an adult. This posits mental health promotion in school as a universal approach for all students within an educational context rather than a health one, focusing on strengths and skills development rather than deficits and mental health conditions (Cavioni et al., 2021; Cefai et al., 2021). Schools have a role both in improving wellbeing and in reducing symptoms of mental health problems (Petersen et al., 2020) by integrating universal interventions for all school children in wellbeing promotion with targeted interventions for students with mental health issues (Cefai et al., 2021), but this paper is focused on the former, namely how schools may promote wellbeing and thus prevent mental health issues.

One of the key drivers of a universal approach to mental health and wellbeing in schools is the curriculum, with interventions aimed at developing students’ social and emotional competence, resilience and mental health (Cavioni et al., 2020). Numerous universal school-based programmes have been developed to promote mental health in schools, spanning from the early years to high school. Such programmes have been found to be effective in enhancing social and emotional competence, positive attitudes and prosocial behaviour, increasing academic achievement and decreasing mental health issues such as internalising and externalising behaviours (e.g., Durlak et al., 2011; Sklad et al., 2012; Goldberg et al., 2019). These positive impacts have been reported from early years to high school, across various cultural and socio-economic contexts, including children from ethnic minorities, low socio-economic background, and children experiencing social, emotional and mental health difficulties (Durlak et al., 2011; Sklad et al., 2012; Taylor et al., 2017). They have been found to be particularly effective for students from low socio-economic background (Wilson and Lipsey, 2007; Taylor et al., 2017), thus operating as a compensatory mechanism (Wigelsworth et al., 2016). However, programmes which have been found to be effective in one context, such as the United States, did not necessarily have such an impact when implemented in other contexts such as Europe (e.g., Armenta et al., 2011; Little et al., 2012). In their review of studies, Wigelsworth et al. (2016) reported “home” programmes (implemented in the context where they were developed) had a stronger effect than “away” programmes (implemented in a different context), with some programmes showing no impact at all when transferred from one cultural context to another.

This paper presents the findings of a study evaluating a new European school-based programme to promote mental health in school. Promoting Mental Health at School (PROMEHS) is an EU funded project comprising nine partners from seven European countries (2019--2022), coordinated by the University of Milano-Bicocca in Italy^1^. The objective of the project was to design, implement and evaluate a mental health promotion curriculum in schools for students, school staff and parents, leading to the development of an evidence-based, universal programme for schools in Europe. The curriculum seeks to enhance students’ social and emotional learning and resilience, as well as reduce social, emotional, and behavioural difficulties (Cavioni et al., 2020; Grazzani et al., 2022b). The aim of this paper is to investigate the effectiveness of the programme across a number of schools in Europe, making use of a semi-randomised control trial design. More specifically the study examines whether students who completed the programme as part of their mainstream curriculum over a number of months in comparison to the control group, had enhanced social and emotional competence, prosocial behaviour and academic engagement and decreased internalising and externalising behaviour difficulties. The study is based on the teachers’ evaluations. It was hypothesised that:

• Students who attended the PROMEHS activities will have higher levels of social and emotional competences (self-awareness and management, social awareness, relationship skills, and decision-making skills), prosocial behaviour and academic outcomes and lower levels of internalising and externalising difficulties, than students in the control group.
• Disadvantaged students’ such as students with learning difficulties and disability and students from low socio-economic background, will benefit more from the programme when compared to typically developing students.
• The programme will be effective across age/year groups and gender.

## Methodology

A quasi-experimental longitudinal design was used to evaluate the impact of the PROMEHS programme on students’ outcomes by comparing the groups’ outcomes within times (pre-test vs. post-test) and between groups (experimental vs. control group). Each of the six implementation countries in the project (Croatia, Greece, Italy, Latvia, Portugal, and Romania) collected data in their respective languages onto one central Survey Monkey database. Due to the COVID-19 situation, not all teachers were able to do the same number of activities during the school year. Consequently, the number of sessions varied between countries due to health policies in place related to the pandemic. The majority of the 423 implementing teachers (59%) completed 10 or more activities, but 31% completed only 4 or fewer activities.

### The intervention

The PROMEHS programme is a universal intervention which acknowledges the importance of working collaboratively among students, teachers, families, school leaders, community stakeholders and policy-makers. It includes three key domains: the promotion of social and emotional learning (SEL) and resilience, and the prevention of social, emotional and behaviour difficulties. Each theme was further divided into specific topics (see Table 1). SEL consisted of five topics, namely self-awareness, self-management, social awareness, relationship skills, and decision-making. Resilience comprised two topics: dealing with psychosocial challenges and dealing with traumatic experiences; whilst prevention of social, emotional and behaviour problems included addressing internalising difficulties, externalising difficulties and risk behaviour (Grazzani et al., 2022b; see Table 1). The programme includes the following components: (1) training courses and supervisions for teachers; (2) manualised handbooks and guidelines for teachers, students, parents and policy-makers (3); meetings with school leaders and parents. In each trial country, a training support team was set to coordinate activities related to the training courses and supervision of teachers, the translation and adaptation of the handbooks and guidelines; and to organise and lead the meetings for school leaders and parents.

**TABLE 1:** PROMEHS programme’s themes and topics.

Theme	Topic
Theme 1: Promoting social and emotional learning	1) Self-awareness 2) Self management 3) Social awareness 4) Relationship skills 5) Responsible decision making
Theme 2: Promoting resilience	1) Dealing with psychosocial challenges 2) Dealing with traumatic experiences
Theme 3: Preventing social, emotional and behavioural difficulties	1) Dealing with internalising problems 2) Dealing with externalising problems 3) Dealing with risk behaviours

### Training courses and supervision for teachers

Teachers in the experimental condition received 16 h of initial training focused on practical and theoretical knowledge about mental health promotion in the school context as well as tools and materials to implement the activities. Areas addressed in the training included promoting teachers’ own mental health (stress, health and coping, social and emotional competence, resilience), promoting students’ mental health through SEL, resilience, classroom climate, and how to implement the programme in the classroom and with parents.

The training was carried out face to face and/or remotely depending on national COVID-19 health regulations. During the implementation, which lasted over a period of 6 months, teachers also received 9 h of mentoring and monitoring by qualified programme trainers. Implementation was planned to be held face to face, but due to COVID-19 regulations, this was not always possible, with some schools doing the programme or parts of it online. A set of procedures were applied to monitor the quality of the implementation across schools and countries. These included the assessment of programme’s fidelity (the extent to which the implemented intervention corresponds to the originally intended programme), dosage (which refers to how much of the intervention has been delivered), quality (related to how well different programme components have been conducted), participants’ responsiveness (referring to the degree to which the programme stimulates the interest and engagement of participants namely teachers, students and parents), and adaptation (related to changes made in the original programme during implementation.

### Manualised activities and guidelines

The PROMEHS programme consists of seven handbooks that provides multi-year programming for students 3–18 years and their parents, and teachers. Four handbooks (two for kindergarten and primary school teachers and students, and two for middle and high secondary school teachers and students) include step-by-step activities that teachers and students carried out respectively at school, as part of the mainstream curriculum, and at home between students and parents/caregivers. The other three volumes contained guidelines on how to promote mental health for teachers, parents and recommendations for policy-makers. Furthermore, two glossaries (for kindergarten and primary school teachers, and for middle and high secondary teachers) have been created to enhance teachers’ mental health literacy. All materials for teachers, students, parents and policy-makers have been nationally adapted and translated into the seven languages of the countries involved in experimentation (Croatian, English, Greek, Italian, Latvian, Portuguese, and Romanian).

### Meetings with school leaders and parents

A total of 6 h, divided in 3 meetings were carried out in order to support the implementation of PROMEHS at systemic level both for the school leaders and the parents. Meetings were organised on monthly basis from January 2021 onward.

### Participants

The sample comprised a total of 7,789 students (3,964 females) from kindergarten to high school across 6 countries in Europe. This consisted of 4,501 participants in the experimental group and 3,288 in the control group, 2,505 participants attending kindergarten, 2,641 primary school, 2,015 lower secondary school, and 628 high school students (see Table 2). This sample guarantees a maximum margin of error of 1.11% assuming a 95% confidence level. The students were recruited from 434 classrooms in 124 schools in six countries, making use of cluster sampling to select schools by gender and school level. Stratified sampling was used to select the students from several classrooms within the selected schools. The teachers of selected students (423 experimental, 356 control) completed a set of questionnaires twice: before implementation of the programme and once the implementation was completed. The sample size of students in the pre-test was 10,602, while the sample size in the post-test was 7,789, so the retention percentage is 73.5% and the drop-out percentage is 26.5%.

**TABLE 2:** Data composition clustered by country, school level, disadvantage, gender and group.

		Group		
		Experimental	Control	*P*-value
Student Gender	Male	2205 (49.0%)	1620 (49.3%)	0.806
	Female	2296 (51.0%)	1668 (50.7%)	
Disadvantaged*	Yes	661 (14.7%)	495 (15.1%)	0.651
	No	3840 (85.3%)	2793 (84.9%)	
School Level	Kindergarten	1369 (30.4%)	1136 (34.6%)	< 0.001
	Primary	1624 (36.1%)	1017 (30.9%)	
	Lower secondary	1124 (25.0%)	891 (27.1%)	
	Higher secondary	384 (8.5%)	244 (7.4%)	
Country	Croatia	404 (9.0%)	386 (11.7%)	< 0.001
	Greece	423 (9.4%)	356 (10.8%)	
	Italy	1073 (23.8%)	589 (17.9%)	
	Latvia	800 (17.8%)	922 (28.1%)	
	Portugal	906 (20.1%)	538 (16.4%)	
	Romania	895 (19.9%)	497 (15.1%)	
Total Sample Size		4501 (100%)	3288 (100%)	

P-value extracted from Chi Square test. *Students with individual educational needs and and disability, students from low socio-economic status.

The administration of questionnaires was mostly completed online, but in some instances hard copies were used; in such cases the researchers from that particular country then inputted the responses in the electronic data base. An indexing system was used so that all data was anonymised. The data file was accessed only by the project evaluation team led by the University of Malta. Ethical approval was obtained from the respective academic institutions and educational authorities and all participants gave their consent before completing the questionnaires. Participants were free to withdraw from the study at any time, and no monetary or other financial rewards were provided.

### Measures

*Social Skills Improvement System, Social Emotional Learning Edition Brief Scales – Student Form (SSIS-SELb-S)* (Elliott et al., 2020*).* This is a measure of social and emotional learning of students in grades 3–12, completed by teachers, parents and students. It is developed on the five SEL domains, namely self-awareness, self-management, social awareness, relationship skills, and responsible decision making. It consists of 20 items, with each of the five subscales (corresponding to the 5 SEL domains) consisting of 4 items. An example of an item from the Social Awareness subscale is “Shows positive attitude in difficult social situations.” The SSIS-SELb-S has strong reliability, with Cronbach’s alphas of 0.91 for the composite score and 0.67–0.72 across the five subscales (Anthony et al., 2020, 2022). Both the composite score and the subscales were used in this study.

*Strengths and Difficulties Questionnaire (SDQ;* Goodman, 1997*).* The SDQ is a brief questionnaire measuring the mental health of 3–16-year-old children, completed by teachers, parents and students (11+). It consists of 25 items comprising five subscales, namely conduct problems and hyperactivity (together comprising Externalising Difficulties), emotional symptoms and peer relationships problems (together comprising Internalising Difficulties), and the Prosocial Scale. An example of an item from the conduct and hyperactivity subscale is “Often has temper tantrums or hot tempers” The first four subscales (problem subscales) give a Total Difficulty Score. In the present study, the three-factor model was used, namely, Internalising Difficulties, Externalising Difficulties, and Prosocial Behaviour (Goodman et al., 2010). In the original instrument, Cronbach’s alphas were 0.66, 0.76, and 0.66 for Internalising, Externalising, and Prosocial scales, respectively (Goodman et al., 2010).

*Academic outcomes* Teachers also completed three questions examining students’ academic motivation, engagement in learning and academic performance (5 point scale from excellent to poor). An example of an item measuring academic engagement is “Engagement in learning process.” A combined response score was used to measure students’ academic outcomes in this study.

Reliability checks found that the items measuring these scales have satisfactory internal consistency. The SSIS-SEL has strong reliability, with Cronbach’s alphas of 0.948 for the composite score and 0.787–0.861 across the five subscales. The three composite subscales of the SDQ also have satisfactory internal consisten**cy** with Cronbach’s alphas of 0.788, 0.867, and 0.838 for the Internalising, Externalising and Prosocial scales respectively. The three-item academic outcome questionnaire showed excellent internal consistency with Cronbach’s Alpha of 0.951.

The questionnaire included a number of demographic questions about the students’ age, school level, and gender, disadvantage (learning difficulties and disability, low socio-economic background), and country where the study was conducted.

### Analysis

Students were matched by code to combine the pre-test and post-test scores, where only students who had scores in both tests were included in the data set. Missing values were replaced by the mean test item score. The Kolmogorov Smirnov test was used to investigate the shape of the score distribution of each subscale. The internalising and externalising difficulties score distributions were right skewed; while prosocial behaviour, social emotional learning and academic outcomes score distributions were right skewed and did not satisfy the normality assumption. To address this limitation, bootstrap standard errors and confidence intervals were provided to account for intrinsic asymmetry and non-Gaussian trends in the regression model. Unlike parametric approaches, bootstrapping resamples a single dataset to create many simulated samples without making any assumptions for the population distribution. This process enables researchers to calculate standard errors, construct confidence intervals and perform hypothesis testing for various types of sample statistics.

Two general linear models were fitted for each subscale to identify the significant risk factors for the difficulty subscales and the significant promotive factors for the prosocial, social emotional learning and academic outcomes subscales. In the first model, the subscale scores of the whole group were related to phase (post-test, pre-test) and group (experimental, control) and their interaction effect to measure the change in the mean subscale score of the experimental group, compared to the control group. In the second model, the subscale scores of the experimental group were related to a number of explanatory variables (phase, school level, student gender, and disadvantage) to identify the significant risk/promotive factors and rank them by their impact on difficulties, prosocial behaviour, social emotional learning and academic outcomes. These explanatory variables were included in the model fits both as main effects and also as interaction effects with phase, which is denoted by *.

## Findings

### Social emotional learning

Tables 3, 4 show that the increment in the mean self-awareness, self-management, social awareness, relationship skills, and responsible decision-making scores from pre- to post-test were significantly larger for the experimental group compared to the control group (*p* < 0.001). The regression coefficients of the interaction terms show that the programme was most effective in enhancing self-awareness compared to the other four domains. Tables 3, 4 also show that the increment in the mean SEL score (composite of the five domains) from pre- to post-test was significantly larger for the experimental group compared to the control group (*p* < 0.001).

**TABLE 3:** Tests of between-subjects effects for social emotional learning and its five domains (whole group).

	Social emotional learning			Self-awareness			Self-management		
Term	Df	F	*P*-value	Df	F	*P*-value	df	F	*P*-value
Intercept	1	502734.126	< 0.001	1	366307.172	< 0.001	1	363424.9	< 0.001
Group	1	9.623	0.002	1	42.324	< 0.001	1	0.296	0.587
Phase	1	99.351	< 0.001	1	123.126	< 0.001	1	40.520	< 0.001
Group * Phase	1	25.772	< 0.001	1	44.219	< 0.001	1	10.733	< 0.001
Error	15574			15574			15574		

	**Social awareness**			**Relationship skills**			**Responsible decision making**		
**Term**	**Df**	**F**	***P*-value**	**Df**	**F**	***P*-value**	**df**	**F**	***P*-value**

Intercept	1	376977.5	< 0.001	1	435746.2	< 0.001	1	416981.4	< 0.001
Group	1	14.209	< 0.001	1	5.125	< 0.024	1	3.272	0.070
Phase	1	97.168	< 0.001	1	90.323	< 0.001	1	52.741	< 0.001
Group * Phase	1	18.765	< 0.001	1	20.677	< 0.001	1	13.448	< 0.001
Error	15574			15574			15574		

**TABLE 4:** Parameter estimates for social emotional learning and its five domains (whole group).

	Social emotional learning			Self-awareness			Self-management		
Parameter	B	Std. Error	*P*-value	B	Std. Error	*P*-value	B	Std. Error	*P*-value
Intercept	3.083	0.009	< 0.001	2.908	0.011	0.000	3.056	0.011	< 0.000
Group = Experimental	–0.017	0.012	0.163	–0.001	0.014	0.919	–0.039	0.014	0.007
Phase = Post	0.043	0.013	0.001	0.044	0.015	0.003	0.031	0.015	0.042
Group = Experimental * Phase = Post	0.089	0.018	< 0.001	0.130	0.020	< 0.001	0.067	0.020	0.001

	**Social awareness**			**Relationship skills**			**Responsible decision making**		
**Parameter**	**B**	**Std. Error**	***P*-value**	**B**	**Std. Error**	***P*-value**	**B**	**Std. Error**	***P*-value**

Intercept	3.060	0.011	< 0.001	3.142	0.010	< 0.001	3.248	0.011	< 0.001
Group = Experimental	–0.006	0.014	0.691	–0.022	0.014	0.106	–0.019	0.014	0.189
Phase = Post	0.056	0.015	< 0.001	0.048	0.015	0.001	0.036	0.015	0.018
Group = Experimental * Phase = Post	0.088	0.020	< 0.001	0.088	0.019	< 0.001	0.074	0.020	< 0.001

Aliased terms are not displayed.

Tables 5, 6 show that in the experimental group, primary and lower secondary school participants scored significantly higher in SEL than kindergarten and higher secondary school participants (*p* < 0.001). Non-disadvantaged participants have significantly larger mean SEL scores than disadvantaged ones (*p* < 0.001), while female participants scored significantly higher than male participants (*p* < 0.001). Although most of the interaction terms are not significant, it is indicative that the programme had a slightly larger impact in enhancing social emotional learning amongst disadvantaged female students attending kindergarten centres.

**TABLE 5:** Tests of between-subjects effects for social emotional learning (experimental group).

	Social emotional learning		
Terms	df	F	*P*-value
Intercept	1	134305.400	< 0.001
School level	3	35.781	< 0.001
Student gender	1	376.658	< 0.001
Disadvantage	1	535.526	< 0.001
Phase	1	55.121	< 0.001
Disadvantage * Phase	1	0.012	0.913
School Level * Phase	3	1.733	0.158
Student Gender * Phase	1	0.056	0.813
Error	8983		

**TABLE 6:** Parameter estimates for social emotional learning (experimental group).

	Social emotional learning		
Parameter	B	S.E.	*P*-value
Intercept	3.112	0.027	< 0.001
School Level = Kindergarten	0.043	0.030	0.148
School Level = Primary	0.157	0.029	< 0.001
School Level = Lower Secondary	0.177	0.031	< 0.001
Student Gender = Male	–0.210	0.015	< 0.001
Disadvantage = Yes	–0.353	0.021	< 0.001
Phase = Post	0.131	0.039	< 0.001
Disadvantaged = Yes * Phase = Post	–0.003	0.031	0.913
School Level = Kindergarten * Phase = Post	0.024	0.043	0.570
School Level = Primary * Phase = Post	–0.003	0.042	0.951
School Level = Lower Secondary * Phase = Post	–0.043	0.044	0.324
Student Gender = Male * Phase = Post	–0.005	0.022	0.813

Aliased terms are not displayed.

### Mental health (Internalising and Externalising Difficulties and Prosocial Behaviour)

Tables 7, 8 show that the reduction in the mean internalising and externalising difficulties scores as well as the increment in the mean prosocial behaviour scores from pre- to post-test were significantly larger for the experimental group compared to the control group (*p* = 0.006; *p* = 0.006, *p* < 0.001). The regression coefficients of the interaction terms show that the programme was more effective in enhancing prosocial behaviour than reducing internalising and externalising difficulties.

**TABLE 7:** Tests of between-subjects effects for internalising and externalising difficulties and prosocial behaviour (whole group).

	Internalising difficulties			Externalising difficulties			Prosocial behaviour		
Term	df	F	*P*-value	Df	F	*P*-value	df	F	*P*-value
Intercept	1	259530.073	< 0.001	1	192278.1	< 0.001	1	433647.417	< 0.001
Group	1	0.314	0.575	1	0.027	0.870	1	17.103	< 0.001
Phase	1	38.290	< 0.001	1	15.547	< 0.001	1	33.045	< 0.001
Group * Phase	1	7.559	0.006	1	7.498	0.006	1	26.402	< 0.001
Error	15574			15574			15574		

**TABLE 8:** Parameter estimates for internalising and externalising difficulties and prosocial behaviour (whole group).

	Internalising difficulties			Externalising difficulties			Prosocial behaviour		
Parameter	B	Std. Error	*P*-value	B	Std. Error	*P*-value	B	Std. Error	*P*-value
Intercept	1.364	0.006	< 0.001	1.418	0.007	< 0.001	2.461	0.008	< 0.001
Group = Experimental	0.012	0.008	0.122	0.017	0.009	0.069	–0.008	0.011	0.478
Phase = Post	–0.018	0.008	0.024	–0.008	0.010	0.428	0.005	0.011	0.688
Group = Experimental * Phase = Post	–0.029	0.011	0.006	–0.035	0.013	0.006	0.077	0.015	< 0.001

Aliased terms are not displayed.

Tables 9, 10 show that in the experimental group, higher secondary school students have significantly larger mean internalising difficulties scores compared to kindergarten, primary and lower secondary school students (*p* < 0.001). Disadvantaged students have significantly larger mean internalising difficulties scores than non-disadvantaged peers (*p* < 0.001), whilst female students have significantly larger mean internalising difficulties scores than males (*p* = 0.015). Although the interaction terms are not significant, it is indicative that the programme had a slightly larger impact in reducing internalising difficulties amongst disadvantaged female students attending kindergarten and lower secondary schools.

**TABLE 9:** Tests of between-subjects effects for internalising and externalising difficulties and prosocial behaviour (experimental group).

	Internalising difficulties			Externalising difficulties			Prosocial behaviour		
Terms	df	F	*P*-value	Df	F	*P*-value	df	F	*P*-value
Intercept	1	85867.169	< 0.001	1	65222.828	< 0.001	1	115754.7	< 0.001
School Level	3	56.463	< 0.001	3	11.772	< 0.001	3	40.652	< 0.001
Student Gender	1	5.976	0.015	1	657.875	< 0.001	1	338.479	< 0.001
Disadvantage	1	618.453	< 0.001	1	536.438	< 0.001	1	251.092	< 0.001
Phase	1	18.594	< 0.001	1	5.133	0.024	1	35.520	< 0.001
Disadvantage * Phase	1	0.046	0.830	1	0.590	0.442	1	0.091	0.763
School Level * Phase	3	0.072	0.975	3	0.478	0.697	3	2.337	0.072
Student Gender * Phase	1	0.026	0.871	1	0.014	0.906	1	0.102	0.750
Error	8983			8983			8983		

**TABLE 10:** Parameter estimates for internalising and externalising difficulties and prosocial behaviour (experimental group).

	Internalising difficulties			Externalising difficulties			Prosocial behaviour		
Parameter	B	S.E.	*P*-value	B	S.E.	*P*-value	B	S.E.	*P*-value
Intercept	1.447	0.017	0.000	1.278	0.020	< 0.001	2.406	0.024	< 0.001
School Level = Kindergarten	–0.160	0.018	< 0.001	0.042	0.022	0.050	0.144	0.026	< 0.001
School Level = Primary	–0.103	0.018	< 0.001	0.025	0.021	0.232	0.216	0.025	< 0.001
School Level = Lower Secondary	–0.124	0.019	< 0.001	–0.027	0.022	0.226	0.176	0.026	< 0.001
Student Gender = Male	–0.015	0.009	0.107	0.204	0.011	< 0.001	–0.171	0.013	< 0.001
Disadvantage = Yes	0.234	0.013	< 0.001	0.247	0.015	< 0.001	–0.213	0.018	< 0.001
Phase = Post	–0.039	0.024	0.099	–0.015	0.028	0.584	0.109	0.033	0.001
Disadvantaged = Yes * Phase = Post	–0.004	0.019	0.830	0.017	0.022	0.442	0.008	0.026	0.763
School Level = Kindergarten * Phase = Post	–0.005	0.026	0.852	–0.034	0.031	0.276	–0.002	0.037	0.951
School Level = Primary * Phase = Post	0.001	0.025	0.997	–0.025	0.030	0.399	–0.040	0.036	0.268
School Level = Lower Secondary * Phase = Post	–0.007	0.026	0.787	–0.017	0.031	0.594	–0.063	0.038	0.092
Student Gender = Male * Phase = Post	0.002	0.013	0.871	–0.002	0.016	0.906	–0.006	0.019	0.750

Aliased terms are not displayed.

Tables 9, 10 show that in the experimental group, lower secondary school students have significantly smaller mean externalising difficulties scores compared to kindergarten, primary and higher secondary school students (*p* < 0.001). Disadvantaged students have significantly larger mean externalising difficulties scores than non-disadvantaged peers (*p* < 0.001), whilst male students have significantly larger mean externalising difficulties scores than females (*p* < 0.001). Although the interaction terms are not significant, it is indicative that the programme had a slightly larger impact in reducing externalising difficulties amongst disadvantaged male students attending kindergarten and primary schools.

Tables 10, 11 show that in the experimental group, kindergarten, primary and lower secondary school students have significantly larger mean prosocial behaviour scores compared to higher secondary school students (*p* < 0.001). Participants from a non-disadvantaged background have significantly larger mean prosocial behaviour scores than-disadvantaged peers (*p* < 0.001), whilst female participants have significantly larger mean prosocial behaviour scores than males (*p* < 0.001). Although most of the interaction terms are not significant, it is indicative that the programme had a slightly larger impact in enhancing prosocial behaviour amongst disadvantaged female students attending kindergarten and higher secondary schools.

**TABLE 11:** Tests of between-subjects effects for academic outcomes.

Source	df	F	*P*-value
Intercept	1	247895.441	< 0.001
Group	1	15.056	< 0.001
Phase	1	34.730	< 0.001
Group * Phase	1	0.609	0.435
Error	15574		

### Academic outcomes

Tables 11, 12 show that the increment in the mean academic outcomes scores from pre- to post-test was larger for the experimental group compared to the control group; however the difference is not significant (*p* = 0.435).

**TABLE 12:** Parameter estimates for academic outcomes.

Parameter	B	Std. Error	*P*-value
Intercept	3.780	0.017	< 0.001
Group = Experimental	0.048	0.022	0.028
Phase = Post	0.079	0.024	< 0.001
Group = Experimental * Phase = Post	0.024	0.031	0.435

Aliased terms are not displayed.

Tables 13, 14 show that in the experimental group, primary and kindergarten school students have significantly higher academic achievement scores than secondary school participants (*p* < 0.001). Female and non-disadvantaged participants similarly had significantly larger mean academic outcomes scores than male and disadvantaged participants respectively (*p* < 0.001, *p* < 0.001). Although most of the interaction terms are not significant, it is indicative that the programme had a slightly larger impact in improving academic outcomes amongst disadvantaged female students attending kindergarten schools.

**TABLE 13:** Tests of between-subjects effects for academic outcomes.

Source	Df	F	*P*-value
Intercept	1	64389.652	< 0.001
School Level	3	17.597	< 0.001
Student Gender	1	156.044	< 0.001
Disadvantage	1	1283.720	< 0.001
Phase	1	10.622	0.001
Disadvantage * Phase	1	0.114	0.735
School Level * Phase	3	2.770	0.040
Student Gender * Phase	1	0.212	0.645
Error	8983		

**TABLE 14:** Parameter estimates for academic outcomes.

Parameter	B	Std. Error	*P*-value
Intercept	4.022	0.047	< 0.001
School Level = Kindergarten	0.063	0.051	0.223
School Level = Primary	0.153	0.050	0.002
School Level = Lower Secondary	–0.008	0.052	0.885
Student Gender = Male	–0.225	0.026	< 0.001
Disadvantage = Yes	–0.947	0.036	< 0.001
Phase = Post	0.127	0.066	0.056
Disadvantaged = Yes * Phase = Post	0.018	0.052	0.735
School Level = Kindergarten * Phase = Post	0.029	0.073	0.688
School Level = Primary * Phase = Post	–0.079	0.071	0.266
School Level = Lower Secondary * Phase = Post	–0.094	0.074	0.205
Student Gender = Male * Phase = Post	–0.017	0.037	0.645

Aliased terms are not displayed.

## Discussion

Based on teachers’ evaluations, the PROMEHS programme had a positive impact on the socio-emotional competence and mental health of the learners who received the intervention. When compared to the matched peers in the control group, the students in the experimental group had significantly larger pre-post increase in social and emotional competence and prosocial behaviour, and decrease in externalising and internalising problems. This is consistent with the main hypothesis of the study and with international studies that universal, school-based programmes such as PROMEHS have a positive impact on social and emotional competence and prosocial attitudes and behaviours, whilst decreasing internalising and externalising difficulties (Durlak et al., 2011; Sklad et al., 2012; Taylor et al., 2017; Cefai et al., 2018). This study also indicates that adequately trained, resourced and mentored teachers can effectively implement SEL programmes in their classroom (Durlak et al., 2011; Sklad et al., 2012; Cefai et al., 2018; Lester et al., 2020).

If one compares the parameter estimates of the interaction term Group* Phase for SEL, SDQ and academic achievement (Tables 3, 7, 11, respectively), the results show that the strongest impact of the programme was in enhancing social and emotional learning and prosocial behaviour followed by a decrease in internalising and externalising difficulties. This is consistent with existing evidence that SEL programmes have the strongest impact on the development of social and emotional skills followed by prosocial behaviour and internalising and externalising difficulties (Durlak et al., 2011; Cavioni et al., 2020). An examination of the different components of SEL, shows that the programme appears to be most effective in enhancing self-awareness followed by social awareness, relationship skills, decision-making, and self-management. The latter three may require more time to develop since they entail behaviour change in contrast to becoming more self and socially aware. Further research is needed to evaluate the specific SEL competences targeted in SEL programmes besides broad measures of social-emotional competences (Ura et al., 2020; Wigelsworth et al., 2020).

In view of existing literature (e.g., Durlak et al., 2011; Corcoran et al., 2018), it was hypothesised that the programme would also have a positive impact on learners’ academic outcomes such as engagement and achievement. However, no such effect was found in this study. One particular issue may be the way academic achievement was construed and measured in the study. Usually academic achievement is measured on the basis of students’ grades in end of term summative assessment. In the present study, it was based on teachers’ perception of students’ motivation, engagement and performance and measured by a simple three-item questionnaire. The conceptual and particularly methodological issues in how academic outcome was measured may at least in part explain the lack of impact of the programme on academic outcomes. Although the three items showed strong internal consistency, we suggest that future studies will evaluate academic outcomes through a more robust measure of academic motivation, engagement and motivation, as well as students’ grades in formal assessment where applicable. Furthermore, the PROMEHS curriculum has been implemented during the COVID pandemic when the schools were closed for several weeks or months (Hammerstein et al., 2021). This had a cascade negative effect on students’ learning processes which may represent a strong limitation in the effectiveness of the programme on the students’ academic outcomes.

*Promoting Mental Health at School* has been developed as a universal school-based programme, tailored to the European context, with a spiral curriculum addressing the increasing complexity and needs of learners from early years to high school, with different activities for different school levels. Such programmes have been found to have an impact on all learners across individual factors such as age and gender and cultural and geographical background (Durlak et al., 2011; Sklad et al., 2012). The findings in this study are consistent with the existing literature, but there are indications that the programme was more effective with early years and female students (except in externalising difficulties) (Grazzani et al., 2022a). Research does indicate that the early years are the period when children’s behaviour is most malleable and that SEL interventions are more likely to impact behaviour the earlier they start (Durlak et al., 2011; January et al., 2011; Jones et al., 2015).

Universal SEL and mental health programmes have been found to be effective for all learners in the group, including those considered disadvantaged or at risk, such as children from low SES, ethnic/migrant background and children with special educational needs (Durlak et al., 2011; Domitrovich et al., 2017; Sanchez et al., 2018). Various reviews have found that universal interventions are particularly effective for children considered at risk- such children may have lower scores at the pre-test level, and thus the programme is more likely to have an impact than on those who already enjoy relatively higher levels of social and emotional competence and mental health (e.g., Durlak et al., 2011; Weare and Nind, 2011; Sanchez et al., 2018). In this study, we found that the programme was effective for both non-disadvantaged and disadvantaged students. There were some indications that on all outcomes, disadvantaged students may have benefitted more from the programme than their peers, but the difference was only marginal. Furthermore, at post-test, students from disadvantaged background still had lower positive scores and higher negative scores than their more advantaged peers, suggesting that PROMEHS might not have triggered a compensatory process by levelling the pre-post- test difference. Research also indicates that universal interventions that are accompanied by targeted interventions for children and young people at risk are more likely to be effective than universal interventions on their own (Weare and Nind, 2011; Goldberg et al., 2019; Murano et al., 2020).

In the present study, we collected data about students’ disadvantage but did not distinguish between different types of disadvantage such as low SES, ethnicity, or learning difficulties. Further research is needed on how universal programmes such as PROMEHS may be moderated by students’ characteristics, such as race/ethnicity, socioeconomic status, disability, and sexual orientation (Wigelsworth et al., 2020).

One of the limitations of this study was that there might have been insufficient time and dosage for behaviour change to take place, particularly in the cases of learners facing challenges and adversity in their learning and development. Not all implementing schools managed to do all the planned activities due to COVID-19 restrictions and disruptions, with the average number of implemented activities being 8.47, though the majority implemented ten or more sessions. Moreover, some of the activities were held online rather than face to face. The implementation of the whole programme over a longer period of time is more likely to bring a positive change (De Mooij et al., 2020; Grazzani et al., 2022b). This will also make it possible to examine whether there are any sleeper effects with outcomes becoming more apparent and visible following a relatively longer period of time (Sklad et al., 2012). The fact that significant impact was found through the implementation of only partial sections of the programme, suggests that there is potential for a higher impact if it is implemented fully and over an extended period of time. Further evaluation studies thus need to take this into consideration when planning the evaluation as dosage is a key determinant of programme effectiveness. In their recent meta-analysis of social skills training programmes, De Mooij et al. (2020) reported that while limited exposure might compromise programme effectiveness, programmes spread over an extended period of time may lose their effectiveness unless the quality of the implementation is regularly monitored.

In some countries the teacher training was held remotely due to COVID-19 restrictions which might have somewhat impact the quality of the training. Adequate teacher training is related to quality implementation which is a key determining factor in programme effectiveness (Domitrovich et al., 2017; Goldberg et al., 2019). Furthermore in some schools the implementation of the activities had to be held online, which might have impacted the quality of the implementation, such as experiential and collaborative learning.

This paper is focused on teachers’ evaluations of programme effectiveness in bringing positive behaviour change in individual student behaviour at school. Teacher-based assessments are highly useful evaluations of students’ behaviour as it occurs in context (school), comparing individual students’ behaviour with that of the group (OECD, 2019). Students’ behaviour varies across contexts, and different actors may perceive behaviour differently. The views of teachers, students and parents have been found to vary considerably on both the Social Skills Improvement Scale (Gresham et al., 2018) and the Strengths and Difficulties Questionnaire (SDQ) (Goodman, 2001). On the other hand, when evaluating the students, the teachers were also indirectly assessing how effective they were in facilitating the programme competences amongst their students, with the possibility of overestimating impact [see also Malouff and Thorsteinsson (2016) on teacher bias in assessment]. Multi-informant evaluations, including self-evaluation by students and parents’ assessment, provide more useful evaluations for both research and practice purposes (Achenbach, 2018; OECD, 2019; see Cefai et al., in preparation).

Finally, due to the nature and constraints of the project, it was not always possible to allocate the experimental and waiting groups randomly and have the teachers and student blind to the nature of the implementation and assessment. A randomised control trial would help to address the limitations of this quasi-experimental study.

## Conclusion

This study indicates that PROMEHS is a promising universal mental health programme for early years, primary and secondary schools in Europe, particularly in enhancing students’ social and emotional competence and prosocial behaviour and decreasing internalising and externalising behaviours. However, further research is needed to evaluate the effectiveness of the programme building on the strengths of the present evaluation protocol whilst addressing its limitations. Further evaluation studies need to make use of a randomised control trial, longitudinal design with follow up trials, make use of multi-informant evaluations, more reliable tools to measure academic achievement and student disadvantage, ensure longer implementation period, monitor for programme dosage and duration, and investigate programme impact by student characteristics such as socio-economic background, cultural and ethnic background, special and educational needs and disability.

## Data availability statement

The raw data supporting the conclusions of this article will be made available by the authors, without undue reservation.

## Ethics statement

The studies involving human participants were reviewed and approved by the University of Rijeka, Croatia; University of Milano-Bicocca, Italy; University of Patras, Greece; University of Latvia, Latvia; University of Lisbon, Portugal, and Stefan Cel Mare University, Suceava, Romania. Written informed consent to participate in this study was provided by the participants’ legal guardian/next of kin.

## Author contributions

CC coordinated the development and writing of the manuscript and made key contributions to designing the research, interpreting the data, and drafting and revising the manuscript. LC made substantial contribution to the conception and design of the research, data analysis and interpretation, and the drafting and revision of the manuscript. PB made substantial contribution to designing the research, interpreting the data, and drafting and revising the manuscript. IG, VC, EC, and VO contributed to the conception and design of the research study, collection of the data, and the drafting and revision of manuscript. The remaining authors contributed to the first draft of the manuscript and were involved in data collection. All authors read and approved the final manuscript.

## Footnotes

1
www.promehs.org
